# Pathogenic infection of *Macaca nemestrina *with a CCR5-tropic subtype-C simian-human immunodeficiency virus

**DOI:** 10.1186/1742-4690-6-65

**Published:** 2009-07-14

**Authors:** On Ho, Kay Larsen, Patricia Polacino, Yun Li, David Anderson, Ruijiang Song, Ruth M Ruprecht, Shiu-Lok Hu

**Affiliations:** 1 Department of Pharmaceutics, University of Washington, Box 357610, Seattle, Washington 98195, USA; 2 Washington National Primate Research Center, University of Washington, 3000 Western Avenue, Seattle, WA 98121, USA; 3 Dana-Farber Cancer Institute, 44 Binney Street, Boston, MA 02115, USA; 4 Harvard Medical School, 25 Shattuck Street, Boston, MA 02115, USA; 5 Aaron Diamond AIDS Research Center, 455 1st Ave, 7th Floor, New York, NY 10016, USA

## Abstract

**Background:**

Although pig-tailed macaques (*Macaca nemestrina*) have been used in AIDS research for years, less is known about the early immunopathogenic events in this species, as compared to rhesus macaques (*Macaca mulatta*). Similarly, the events in early infection are well-characterized for simian immunodeficiency viruses (SIV), but less so for chimeric simian-human immunodeficiency viruses (SHIV), although the latter have been widely used in HIV vaccine studies. Here, we report the consequences of intrarectal infection with a CCR5-tropic clade C SHIV-1157ipd3N4 in pig-tailed macaques.

**Results:**

Plasma and cell-associated virus was detectable in peripheral blood and intestinal tissues of all four pig-tailed macaques following intrarectal inoculation with SHIV-1157ipd3N4. We also observed a rapid and irreversible loss of CD4^+ ^T cells at multiple mucosal sites, resulting in a marked decrease of CD4:CD8 T cell ratios 0.5–4 weeks after inoculation. This depletion targeted subsets of CD4^+ ^T cells expressing the CCR5 coreceptor and having a CD28^-^CD95^+ ^effector memory phenotype, consistent with the R5-tropism of SHIV-1157ipd3N4. All three animals that were studied beyond the acute phase seroconverted as early as week 4, with two developing cross-clade neutralizing antibody responses by week 24. These two animals also demonstrated persistent plasma viremia for >48 weeks. One of these animals developed AIDS, as shown by peripheral blood CD4^+ ^T-cell depletion starting at 20 weeks post inoculation.

**Conclusion:**

These findings indicate that SHIV-1157ipd3N4-induced pathogenesis in pig-tailed macaques followed a similar course as SIV-infected rhesus macaques. Thus, R5 SHIV-C-infection of pig-tailed macaques could provide a useful and relevant model for AIDS vaccine and pathogenesis research.

## Background

The research of AIDS pathogenesis has been facilitated by the use of Asian macaques known to develop AIDS-like diseases from lentivirus infection, including rhesus (*M. mulatta*), cynomolgus (*M. fascicularis*), and pig-tailed (*M. nemestrina*) macaques [1-11]. Studies in rhesus macaques have provided extensive insight into the biology of disease-susceptible animals to advance ongoing efforts towards developing an effective human AIDS vaccine. On the other hand, much less is known about the early events after lentiviral infection in other macaque species, including pig-tailed macaques.

The species/subspecies of macaques used in a study can be a significant determinant of viral infectivity and disease susceptibility. For example, in a comparative study of Asian macaques infected intravenously with simian immunodeficiency virus (SIV) or simian-human immunodeficiency virus (SHIV) strains, SIVmac251 or SHIV89.6P, Reimann et al. found lower plasma viral loads, higher levels of peripheral CD4^+ ^T cells, and higher survival rates in cynomolgus and Chinese rhesus, compared to similarly infected Indian rhesus [12]. Interestingly, ten Haaft et al. reported contrasting findings in cynomolgus vs. Indian rhesus infected intravenously or via select mucosal routes [13]. Their study showed that while cynomolgus macaques had lower steady-state viral loads after SIV infection, there was no such difference after SHIV89.6P infection. Consistent with the Reimann et al. report above, Ling et al. also showed a differential response to lentiviral infection at the subspecies level. Compared to their Indian counterparts, Chinese rhesus infected with SIVmac239 had lower plasma viral loads in acute infection, maintained lower setpoint plasma viremia, and experienced less severe depletion of intestinal CD4^+ ^effector cells, all of which resulted in better clinical outcomes [14]. However, Burdo et al. found that serial passage of SIVmac128 in Chinese rhesus resulted in increased steady-state viral loads as compared to animals infected with the virus derived from Indian monkeys, implying that host adaptation plays an important role in viral fitness and pathogenicity [15].

Taken together, these findings suggest that the efforts to develop an AIDS vaccine may be well served by examining a diverse range of antiviral responses and disease susceptibilities in different animal models. Pig-tailed macaques are of particular interest for several reasons. First, despite sharing a common ancestor, pig-tailed macaques are more distantly related to cynomolgus and rhesus macaques than the latter species are to each other [16,17]. This evolutionary distance may have genetic implications affecting components of the adaptive immune response, including T-cell receptor diversity and major histocompatibility complex (MHC) molecules [18,19]. Second, pig-tailed macaques are defective in a restriction factor TRIM5α [20] used by rhesus macaques to inhibit replication by certain retroviruses, such as HIV-1 [21]. Pig-tailed macaques have previously been shown to be susceptible to infection by HIV-1 [22,23] and recently, by simian-tropic (st)HIV-1 strains [24]. Third, evidence exists indicating that pig-tails are more susceptible to lentivirus-induced disease. In a comparative study of pig-tailed and rhesus macaques infected with SHIV_SF162P4_, Polacino et al. found higher peak and setpoint viral loads in pig-tailed macaques despite similar infectivity between the two species, demonstrating that pig-tails were less able to control infection [25]. This finding was consistent with an early report by Rosenberg et al., who found that SIV_PBj-14_-infected pig-tailed macaques were more susceptible to death resulting from gastrointestinal distress than their rhesus counterparts [26]. Similarly, other studies have documented persistent infection, CD4^+ ^T cell depletion, and/or development of AIDS-like diseases in pig-tails, but not rhesus, infected with HIV-2 primary isolates [27-29]. Thus, based on their increased susceptibility to HIV infection and to lentivirus-induced disease, compared to rhesus, pig-tailed macaques may be a useful animal model for addressing the diverse responses to HIV-1 infection in humans.

Elucidation of the immunopathogenic events in mucosa-associated lymphoid tissue (MALT) has been a major advance in AIDS research in the last ten years [30,31]. Dramatic and irreversible depletion of CD4^+ ^T cells at multiple mucosal sites occurs early after SIV infection [32-42]. Furthermore, the virus specifically targets CCR5^+ ^and activated memory CD4^+ ^T-cells [35,37,40-45] comprising the majority of total lymphocytes found in MALT, especially in the intestine, the largest immunologic organ in the body [46,47]. In contrast, these subsets represent small numbers of circulating CD4^+ ^lymphocytes in blood, lymph nodes, and other secondary lymphoid tissues. Consequently, depletion of CD4^+ ^T cells in these tissues is not as dramatic as in the mucosal compartment during acute infection [32,35,37-39,42,48]. Thus, monitoring mucosal CD4^+ ^T cells may provide important insight into lentivirus-induced immunopathogenesis. However, compared to the extensive knowledge accumulated from rhesus studies, less is known about mucosal pathogenic events in pig-tailed macaques during early infection.

The rapid depletion of CD4^+ ^T cells observed in the MALT of SIV-infected macaques contrasts with the depletion observed in peripheral blood of macaques infected with the first-generation of SHIVs, such as SHIV-HXBc2 and SHIV89.6P. This discrepancy most likely reflects the CXCR4-tropism of these SHIVs vs. the CCR5-tropism of SIV [49,50]. As most of the transmitted viruses in sexual and maternal-infant HIV-1 infection are CCR5-tropic, the use of R5 SHIVs may be more biologically relevant in preclinical vaccine studies [51]. Furthermore, currently available SHIVs are predominantly derived from subtype B isolates of HIV-1 [52-57], whereas the majority of global infections results from subtype C virus transmission [51]. Recently, Song et al. reported the construction of a CCR5-tropic subtype C SHIV-1157ipd3N4 (also referred to as SHIV-C for short), which has been shown to be highly replication-competent and mucosally transmissible in Indian- and Chinese-origin rhesus macaques [58]. Three of five rhesus infected with parental versions of SHIV-1157ipd3N4 progressed to AIDS within 2–5 years postexposure [59]. Two of the 13 rhesus monkeys infected with SHIV-1157ipd3N4 also progressed to AIDS within 80–100 weeks (Chenine et al., unpublished data). This pathogenic R5-tropic SHIV-C may therefore represent an important tool for pathogenesis study of primate lentiviruses and preclinical evaluation of candidate HIV vaccines. In the present study, we evaluated the infectivity and pathogenicity of SHIV-1157ipd3N4 in pig-tailed macaques to determine its potential role as an alternative challenge model in future AIDS vaccine studies.

## Results and discussion

### SHIV-1157ipd3N4 infection in pig-tailed macaques

**Figure 1:**
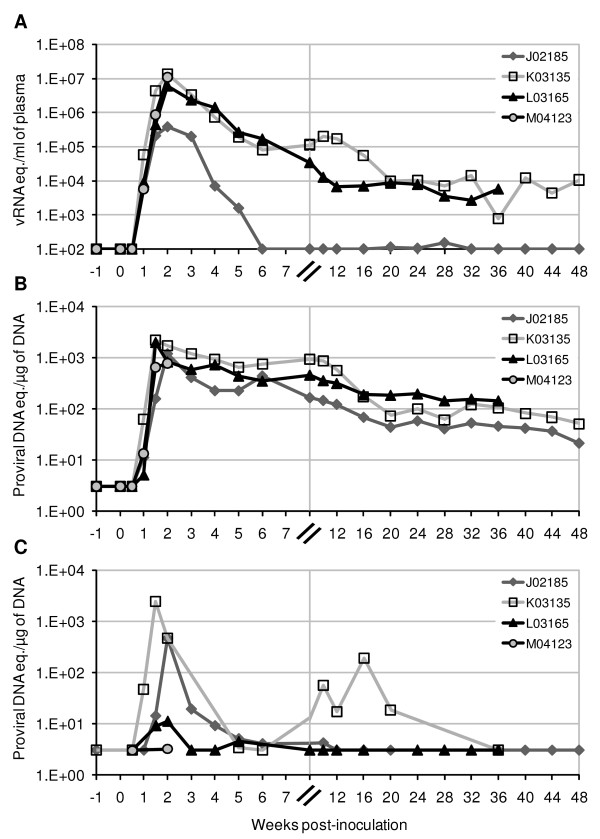
**Plasma and cell-associated viral loads in pig-tailed macaques infected with SHIV-1157ipd3N4**. Viral RNA loads were measured in plasma (A), and proviral cDNA loads in total mononuclear cells isolated from peripheral blood (B) and duodenum (C). To distinguish data points in the early stages of infection, a scale break (//) corresponding to week 8 after inoculation was inserted into the x-axis (same for subsequent figures).

All four pig-tailed macaques inoculated intrarectally with SHIV-1157ipd3N4 were susceptible to infection and showed peak plasma viral loads averaging 7.6 ± 5.8 × 10^6 ^viral RNA copies/ml by 2 weeks post-inoculation (p.i.) (Fig. 1A). At this time, macaque M04123 died due to complications of the intestinal biopsy procedure. Its terminal plasma viral load was 1.1 × 10^7 ^copies/ml. Plasma viremia persisted in two of the three remaining animals, with levels ranging from 7 × 10^3 ^to 2 × 10^5 ^copies/ml of plasma. In contrast, virus replication was controlled below the level of quantification (100 copies/ml) in macaque J02185 by week 6 following inoculation. Similar kinetics of infectivity were observed in peripheral blood and mucosal mononuclear cells (PBMC and MMC), where mean viral loads peaked by 1.5–2 weeks p.i. (1.5 ± 0.6 × 10^3 ^and 0.3 ± 0.2 × 10^3 ^copies/μg of DNA, respectively; Fig. 1B–C). After the initial peak of viremia, viral load in PBMC persisted in all three macaques within a range of 21 to 915 copies/μg of DNA (Fig. 1B). In the duodenum, viral load in MMC was below detection by week 6 p.i., except in macaque K03135 that showed elevated levels of virus between weeks 10 and 16 p.i. (Fig. 1C).

The fact that all four pig-tailed macaques became infected after inoculation with SHIV-1157ipd3N4 confirmed the susceptibility of this species to infection by this virus, which was propagated and studied only in rhesus monkeys [58]. Peak viral loads from the four infected pig-tailed macaques approached the lower range reported for Indian rhesus, and within the range for Chinese rhesus [58] (Fig. 1A). Plasma virus also peaked at the same time in both species (2 weeks p.i.). Viremia persisted in 2/3 pig-tails during the year-long study, similar to results reported for rhesus monkeys (3/5 and 3/8, respectively, for Chinese and Indian rhesus) [58] (Fig. 1A).

### Early and severe SHIV-1157ipd3N4-induced mucosal immunopathogenesis

**Figure 2:**
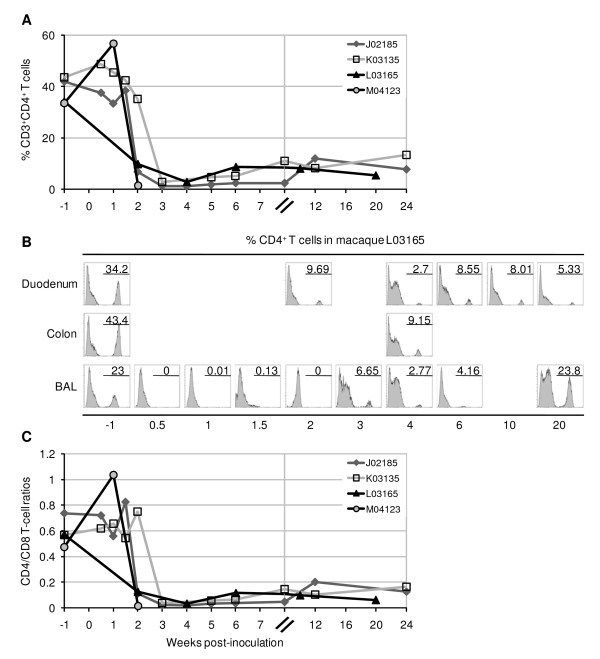
**Mucosal CD4^+ ^T-cell depletion due to SHIV-1157ipd3N4 infection**. (A) Total lymphocytes isolated from duodenal biopsies from infected pig-tailed macaques were analyzed by flow cytometry for CD3^+^CD4^+ ^T cells. CD4^+ ^T-cell percentages were obtained by gating on CD3^+ ^T cells and then lymphocytes. (B) Histogram plots showing a comparison of CD4^+ ^T-cell percentages in mucosal tissues of macaque L03165. Duodenal and colonic biopsies, and BAL samples, were taken concurrently at the specified timepoints pre- and post-inoculation. (C) CD4:CD8 ratios in the duodenum were generated by using the percentages of total CD4^+ ^and CD8^+ ^T cells.

To examine the effect of R5 SHIV-C infection on mucosal CD4^+ ^T cells, especially during the early stages after virus inoculation, we performed a longitudinal analysis of CD3^+^CD4^+ ^T lymphocytes by flow cytometry. As early as 2 weeks p.i., CD3^+^CD4^+ ^T cells in the duodenum had significantly decreased from a pre-inoculation level of 38.3% to 13.3% (standard deviation of 5.2% and 15%, respectively) (Fig. 2A). By 3–4 weeks p.i., only 2.2 ± 1% of CD3^+^CD4^+ ^T lymphocytes were detectable in the duodenum of three animals, reflecting a dramatic depletion of 92–97% of the total CD4^+ ^T-cell population in the duodenal mucosa. Notably, despite the nearly undetectable plasma and MMC viral load in macaque J01285 by 6 weeks p.i., the ability to control virus replication did not appear to lessen the depletion of intestinal CD4^+ ^T cells in this animal (Fig. 1A and 1C; and Fig. 2A). In fact, J02185 showed the highest degree of CD4^+ ^T-cell depletion in the duodenum at 97% by 4 weeks p.i. For all three animals, the percentages of CD3^+^CD4^+ ^T cells in the duodenum slightly recovered over the course of 24 weeks to levels that did not exceed 11.3 ± 2.5%, or approximately 28% of pre-inoculation levels (Fig. 2A).

The severe loss of CD3^+^CD4^+ ^T cells was also found in other mucosal tissues, including the colon and the lung, the latter accessible by bronchoalveolar lavage (BAL) sampling (Fig. 2B). Similar to the duodenum, depletion of CD4^+ ^T cells was not observed in the colon at week 1 p.i. in two macaques (data not shown). By week 4 p.i., CD4^+ ^T-cell levels had decreased from 43.4% to 9.2%, or 79% from pre-existing levels in macaque L03165. The elimination of CD4^+ ^T lymphocytes was more severe and rapid in lung mucosa, where CD3^+^CD4^+ ^T cells were undetectable by 3 days p.i. In fact, BAL CD4^+ ^T cells remained at undetectable or nearly undetectable levels for 1–2 weeks after virus inoculation in two macaques (Fig. 2B; and data not shown). However, in at least one macaque (L03165), the percentage of CD3^+^CD4^+ ^T cells in the BAL returned to pre-inoculation levels by week 20 p.i. (Fig. 2B).

As a result of the profound depletion of CD4^+ ^T cells in the mucosal tissues, we observed a striking decrease of CD4:CD8 T cell ratios during acute infection (Fig. 2C). By 2–4 weeks p.i., the T-cell ratios in the duodenum had decreased nearly 23-fold, from a pre-inoculation range of 0.47–0.74 to a post-inoculation range of 0.016–0.037. The decrease in the duodenal T-cell ratios largely persisted throughout the course of infection, with recovery in CD3^+^CD4^+ ^T-cell levels resulting in only minimal increases in the CD4:CD8 ratios, which did not exceed 0.18. In macaque L03165, the massive elimination of BAL CD4^+ ^T cells as early as 3 days p.i. resulted in a marked decrease of CD4:CD8 T-cell ratios up to 2 weeks p.i., dropping from a pre-inoculation ratio of 0.43 to a range of 0–0.003 after infection (data not shown).

To our knowledge, this is the first report of a prospective analysis of the immunopathogenesis in multiple mucosal compartments during infection with a R5 SHIV in pig-tailed macaques. Chen et al. found that pig-tailed macaques infected with a R5-tropic clade C SHIV_CHN19P4 _were significantly depleted of jejunal CD4^+ ^T cells at 2 weeks p.i., with no remarkable change in immune activation or proliferation of CD4^+ ^gut lymphocytes, as measured by CD25 and Ki67 staining, respectively [60,61]. Chase et al. reported that ileal CCR5^+^CD4^+ ^T cells of pig-tails inoculated with an immunosuppressive viral strain SIV/ΔB670 together with a macrophage-tropic molecular clone SIV/17E-Fr dropped from average uninfected levels of 44% to 8% by 2 weeks p.i. [62]. A significant decrease in CD25^hi ^cells and an increase in Ki67^+ ^cells were also observed in CD4^+ ^gut lymphocytes from the SIV-infected animals [62]. Our data confirm and extend these previous findings that gut immunopathogenesis is a hallmark of early R5-tropic SIV/SHIV infection in pig-tailed macaques.

The results reported here also indicate that R5 SHIV-C-induced mucosal pathogenesis in pig-tails followed a similar course as described in SIV-infected rhesus. These studies demonstrated profound CD4^+ ^T-cell losses, nearly complete in some cases, by days 10–21 after infection [32-42]. There is no discernable difference in the depletion of CD4^+ ^T lymphocytes within intestinal and lung mucosa in pig-tails, with regard to the kinetics or severity of the depletion, compared to these previous reports in rhesus. Notably, the elimination of mucosal CD4^+ ^T cells in pig-tailed macaques was often followed by the partial or limited return of these populations over the course of the study period (Fig. 2A–B). In fact, the partial recovery of CD4^+ ^T cells in the gut of SIVmac251-infected rhesus macaques has been documented by Veazey et al., who showed intestinal CD3^+^CD4^+ ^T cells increased up to 20% by week 5 p.i. in a few infected animals [40]. Similarly, Okoye et al. reported that BAL CD4^+ ^T-cell levels from SIVmac239-infected rhesus initially recovered after early depletion up to approximately 14 weeks p.i., then decreased progressively thereafter [63]. The mechanism underlying the recovery of mucosal CD4^+ ^T cells after acute infection remains to be defined, although there is evidence identifying the majority of repopulating intestinal CD4^+ ^T cells as naïve [40,42]. While the rebound of mucosal CD4^+ ^T cells appeared to be partial and/or transient, depletion of this cell population in early SHIV infection was sustained during the course of infection (Fig. 2A–B). Moreover, peak infection of intestinal mononuclear cells at 1.5–2 weeks p.i. coincided with the onset of CD4^+ ^T-cell depletion, consistent with previous findings [33,35,38,39,44,46,61,64,65] (Figs. 1C and 2A). Thus, the availability of target cells is a critical determinant of mucosal immunopathogenesis.

### Acute SHIV-1157ipd3N4 infection results in specific elimination of mucosal CCR5^+ ^and effector memory CD4^+ ^T lymphocytes

**Figure 3:**
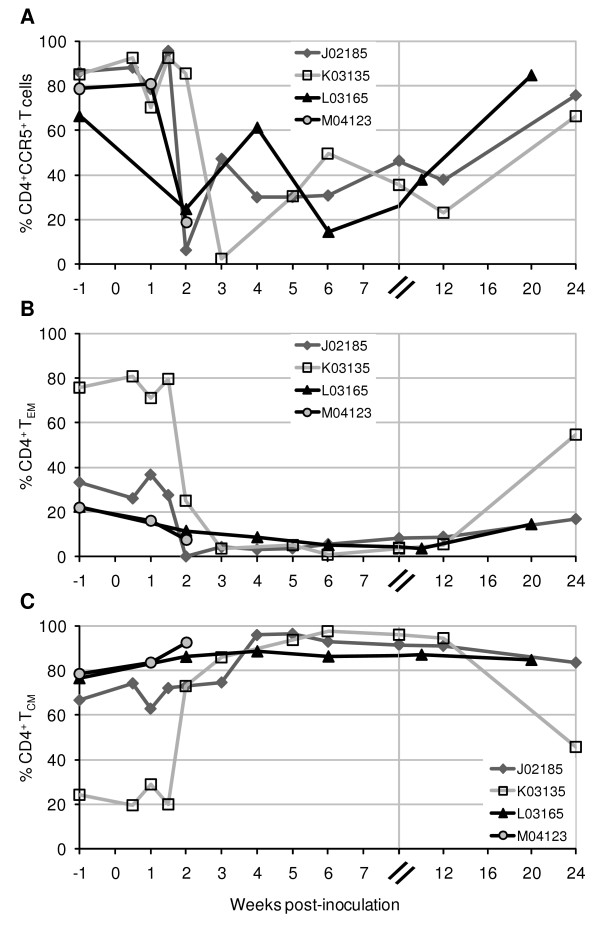
**Selective loss of intestinal CCR5^+ ^and effector memory CD4^+ ^T cells during early R5 SHIV-C infection**. Total lymphocytes isolated from duodenal biopsies from SHIV-1157ipd3N4-infected pig-tailed macaques were analyzed by flow cytometry for CD4^+ ^T-cell subsets based on expression of (A) CCR5^+ ^(B) CD28^-^CD95^+ ^(T_EM_) and (C) CD28^+^CD95^+ ^(T_CM_). Percentages of subsets were obtained by gating on CD3^+ ^T cells, lymphocytes, and then CD4^+ ^T cells.

The selective targeting of CCR5^+ ^and effector memory CD4^+ ^T cells in the mucosal compartment has been well documented in SIV-infected rhesus macaques [35,37,40-45], yet less is known about these subsets in pig-tailed macaques during infection. We therefore examined mucosal CD4^+ ^T cells for CCR5 and effector memory markers. By 2–3 weeks p.i., we found that a large decrease of duodenal CD4^+^CCR5^+ ^T cells had occurred in all four SHIV-1157ipd3N4-infected pig-tailed macaques. Percentages of CCR5-expressing T cells dropped from 79% in uninfected animals to 13% by 2–3 weeks p.i. (standard deviation of 9% and 10.5%, respectively), or approximately a decrease of 82 ± 15.8% from pre-inoculation levels (Fig. 3A). After the initial early depletion, the percentages of CD4^+^CCR5^+ ^T cells in three macaques showed fluctuations before increasing during the later stages of the study period. By 20–24 weeks p.i., the percentages of CD4^+^CCR5^+ ^T cells had recovered to 98 ± 26% of pre-inoculation numbers (Fig. 3A). It is unknown whether this level of recovery was transient, based on a singular measurement after week 12, or influenced by the small CD4^+ ^T-cell population remaining (8.8 ± 4.2%) at 20–24 weeks p.i. Yet, the fact that this increase occurred in all three animals indicates the recovery of CD4^+^CCR5^+ ^T cells during this time was consistent (Fig. 3A).

A substantial reduction of CD4^+ ^effector memory T cells (T_EM_), identified by their CD28^-^CD95^+ ^phenotype, was also seen in the duodenum of all four infected pig-tails. By 2–4 weeks p.i., these numbers fell from 33.2% to 0% in J02185; 75.6% to 3.6% in K03135; 22.2% to 8.6% in L03165; and 21.9% to 7.4% in M04123 (Fig. 3B). On average, CD4^+ ^T_EM _dropped from 38.2% to 4.9% (standard deviation of 25.5% and 3.9%, respectively), reflecting an 80.7 ± 19.7% decrease of pre-existing populations. CD4^+ ^T_EM _cells were undetectable by flow cytometry at 2 weeks p.i. in macaque J02185, the same animal whose CD4^+ ^T cells were 97% depleted by week 4 p.i. (Figs. 2A and 3B). By 20–24 weeks p.i., the numbers of CD4^+ ^T_EM _in all three macaques followed beyond the acute phase had recovered to 62.4 ± 11.3% of their pre-inoculation levels, despite only a 28% recovery in total CD4^+ ^T cells (Figs. 2A and 3B). The coincident increase of central memory CD4^+ ^T cells (T_CM_) beginning at 2 weeks p.i., when the effector memory subset was profoundly depleted (Fig. 3C), suggests a homeostatic mechanism whereby the T_EM _are derived from the proliferation and differentiation of the T_CM _population [63]. Thereafter, T_CM _levels reached a plateau in all three animals until week 12 p.i., at which time this cell population decreased in K03135.

Our results indicate that SHIV-1157ipd3N4 induced a similar immunopathogenesis in pig-tails as SIV in rhesus, based on the R5-tropism of both viruses. Rhesus macaques showed a dramatic decline of mucosal CD4^+ ^T cells with a CCR5^+ ^memory phenotype at 11–28 days following infection [35,37,40-45] The specific targeting of mucosal CCR5 and effector memory CD4^+ ^T cell subsets is consistent with the elevated levels of CCR5 on activated memory T cells [32,46,47], and the predominance of an activated/memory phenotype in mucosal tissue [30,31]. In fact, rhesus studies have reported that large numbers of CCR5^+ ^target cells reside at mucosal sites, including the gut and lung, where approximately 50–90% of CD4^+ ^T cells express CCR5 [30-32,35,40,41,44,66]. We found comparable levels of CCR5^+^CD4^+ ^T cells in our analysis of mucosal tissues from uninfected pig-tailed macaques, including the duodenum (79 ± 9%), colon (63 ± 10%), and BAL (98 ± 0.4%), the latter reported for two of four animals. Therefore, as reported in SIV-infected rhesus, the mucosa provides a critical reservoir of CD4^+ ^target cells for R5-specific SHIV-C infection in pig-tails as demonstrated by the sharp decrease of CCR5^+ ^and T_EM _cells by 2 weeks p.i (Fig. 3A–B).

The basis for the apparent increase of intestinal CCR5^+ ^and T_EM _cells after initial depletion (Fig. 3A–B) is not well understood. As the percentages of the subsets are based on total CD4^+ ^T numbers, it follows that proportional increases in CCR5^+ ^and T_EM _cells concurrent with severe CD4^+ ^T-cell losses during acute and chronic infection (Fig. 2A) may result in the apparent "recovery" of the subset populations [42]. Further, Veazey et al. observed at 2 weeks p.i. that more than 50% of the residual intestinal CD4^+ ^T cells in a few SIV-infected rhesus were CCR5^+ ^and naive (CD45RA^HI^) [40]. At 4–6 months p.i. most residual CD4^+ ^T cells in the gut were naïve, but lacked CCR5 expression [40]. Thus, while it is possible that the "recovery" of CCR5^+ ^and memory subsets may represent actual residual cells which remained and expanded, these populations may also be derived from naive cells newly formed and recruited to the mucosa early after infection [40,42].

### Loss of specific CD4^+ ^T cell subsets during acute SHIV-1157ipd3N4 infection also occurs in peripheral blood

**Figure 4:**
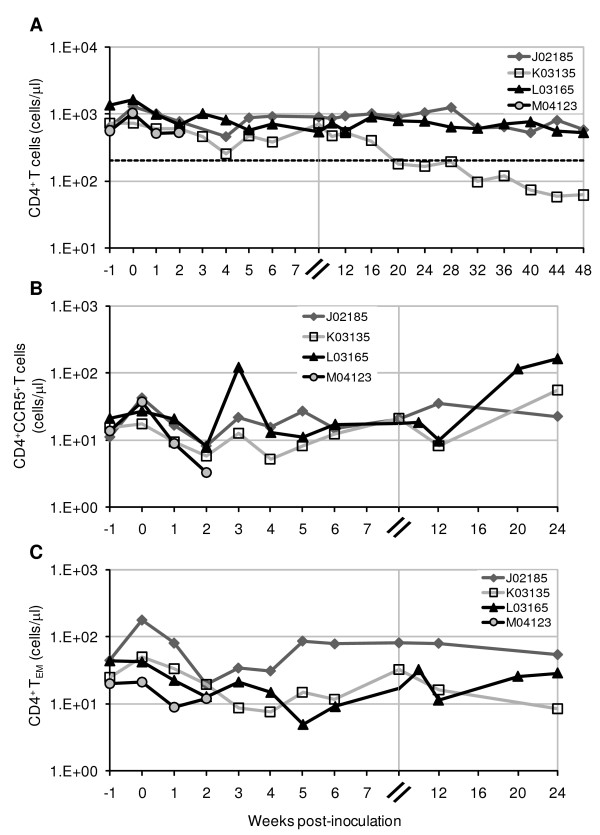
**Selective loss of CD4^+ ^T-cell subsets in the peripheral blood of R5 SHIV-C-infected pig-tailed macaques**. Flow cytometric evaluation of (A) total CD4^+ ^T cells and subsets based on expression of (B) CCR5^+ ^and (C) CD28^-^CD95^+ ^(T_EM_) was done using whole blood (for total CD4^+ ^T cells) or total lymphocytes isolated from peripheral blood (for subsets). Analysis was performed by gating first on lymphocytes and then CD3^+^CD4^+ ^T cells. Total absolute counts of CD4^+ ^T cells were calculated by multiplying the percentage of CD3^+^CD4^+ ^T cells by the number of total lymphocytes/ml from complete blood count (CBC) analysis. Further multiplication of the percentage of cells expressing CCR5^+ ^or CD28^-^CD95^+ ^was done to calculate the absolute counts of the subsets. The dashed line in (A) indicates 200 cells/μl, the threshold level defining human AIDS.

A comparatively smaller, but detectable, decrease in the absolute number of CD3^+^CD4^+ ^T-cells was also observed in peripheral blood at early timepoints following virus inoculation, dropping 12 ± 29% and 44 ± 19% from pre-existing levels, at 2 and 4 weeks p.i., respectively (Fig. 4A). Thereafter, absolute peripheral CD4^+ ^T-cell counts remained relatively stable in two of three animals, ranging from 519 to 1,257 cells/μl for the duration of the study period. CD4^+ ^T cell numbers dropped below 200 cells/μl in macaque K03135 at week 20 p.i. and have since shown a progressive decline at all subsequent timepoints, indicating progression to AIDS. Between weeks 10–16 p.i., animal K03135 showed a pronounced increase in viral load in the duodenum (Fig. 1C). The basis for this increase is not clear, but may reflect immune escape or local reactivation of latent viruses [67-69]. There were also no overt clinical signs that correlated with this distinct viral peak. However, it is notable that this increase in MMC viral load occurred at the same time as the increase in plasma viral load (Fig. 1A), and just before the decline of CD4^+ ^T-cells in peripheral blood (Fig. 4A).

We also observed decreases in the absolute counts of CCR5^+ ^and CD28^-^CD95^+ ^subsets in peripheral blood CD4^+ ^T cells from all four pig-tailed macaques in early R5 SHIV-C infection (Fig. 4B–C). By 2–3 weeks p.i., CCR5-expressing T cells dropped from 15.2 to 6.2 cells/μl (standard deviation of 4.1 and 2.3 cells/μl, respectively), or 56.9 ± 21.6% from pre-challenge levels (Fig. 4B). Similarly, CD4^+ ^T_EM _fell from 32.9 to 13.1 cells/μl (standard deviation of 12.5 and 4.4 cells/μl, respectively), reflecting a loss of 58 ± 13.7% in this population (Fig. 4C). However, compared to the duodenum, substantially lower levels of these subsets were found in the peripheral blood of uninfected pig-tails (for CCR5^+^, 79 ± 9% vs. 2.8 ± 0.3%; for CD28^-^CD95^+^, 38.2 ± 25.5% vs. 4.5 ± 1.7%) (Fig. 3A–B; and data not shown). Similarly low levels of peripheral blood CD4^+ ^T cells expressing CCR5 and having an effector memory phenotype (approximately 3–16%) have been reported in rhesus macaques [30,31,35,40,41,66]. Thus, consistent with previous findings in rhesus, the small numbers of pre-existing peripheral CCR5^+ ^and T_EM _cells in pig-tailed macaques precluded dramatic changes to the total absolute CD4^+ ^T-cell count, despite substantial losses to the population subsets due to SHIV-C infection (Fig. 4).

**Figure 5:**
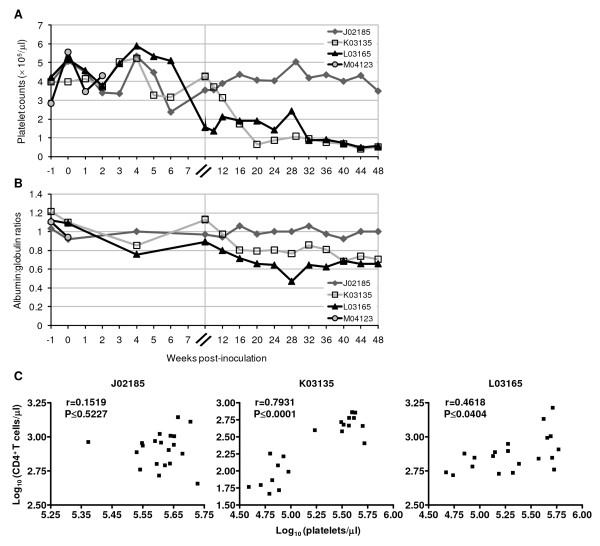
**Thrombocytopenia and hematological changes in SHIV-C-infected pig-tailed macaques**. Blood samples were collected at the indicated times following SHIV-C inoculation and analyzed for (A) platelet counts, (B) albumin:globulin ratios, and (C) correlation between platelet and peripheral CD4^+ ^T-cell counts. Note the different scales along the x- and y-axes in (C). Spearman's correlation coefficient and statistical significance (r- and p-values, respectively) were calculated with Prizm 4 (GraphPad Software, Inc).

Macaque L03165 died under anesthesia during a mucosal sampling procedure at 48 weeks p.i. This animal had shown persistent viremia, but otherwise normal peripheral CD4^+ ^T-cell levels (Figs. 1A and 4A). Necropsy revealed a near-occlusive pulmonary arterial thrombus. The clinical history of L03165 indicated a dramatically reduced platelet count and a moderate decrease in the albumin:globulin protein ratio (Fig. 5A–B). A reduced platelet count has also been documented in macaque K03135, the only animal to have developed peripheral CD4^+ ^T-cell lymphopenia (Fig. 4A; and Fig. 5A and 5C).

Findings reported here demonstrate the pathogenic potential of SHIV-1157ipd3N4 infection in pig-tailed macaques. The differential clinical responses in J02185, K03135, and L03165 (Figs. 1A, C, and 4A) suggest a spectrum of disease courses is possible from SHIV-C infections, consistent with previous findings in SIV and R5 SHIV strains, and the outbred nature of the animals studied. Peripheral lymphopenia and AIDS-defining events have also been documented in an Indian rhesus infected with SHIV-1157ipd, a late-stage biological isolate from which SHIV-1157ipd3N4 was directly derived [58,59]. Additionally, long-term monitoring of SHIV-1157ipd3N4 infection in rhesus has shown AIDS progression in two animals (Chenine et al., unpublished data). Song et al. reported relatively stable absolute peripheral CD4^+ ^T-cell counts in all Indian- and Chinese-origin rhesus acutely infected with SHIV-1157ipd3N4 and followed for 12 weeks [58]. They also found that 43–66% of peripheral CD4^+ ^T_CM _was depleted in two of five Indian rhesus at 8 weeks p.i. Compared to pre-existing levels at week 0, we found a similar 39–59% decrease of peripheral CD4^+ ^T_CM _in pig-tails at 2 weeks p.i.; by 8 weeks p.i., this decrease was 5–28%, reflecting the partial recovery of these cells in blood (data not shown). Interestingly, we observed an association between low pre-inoculation counts of peripheral CD4^+ ^T_CM _and progression to SHIV-C-induced disease in one animal (K03135) (data not shown), consistent with previous findings in SIVmac251-infected pig-tailed macaques [70]. Rhesus monkeys infected with another pathogenic R5 SHIV-C, SHIV-2873Nip, which was constructed from the backbone of SHIV-1157ipd3N4, also demonstrated a loss of peripheral CD4^+ ^memory T cells, along with depletion of gut CD4^+ ^T lymphocytes [71].

### Antibody-mediated immune responses to SHIV-1157ipd3N4 infection

**Figure 6:**
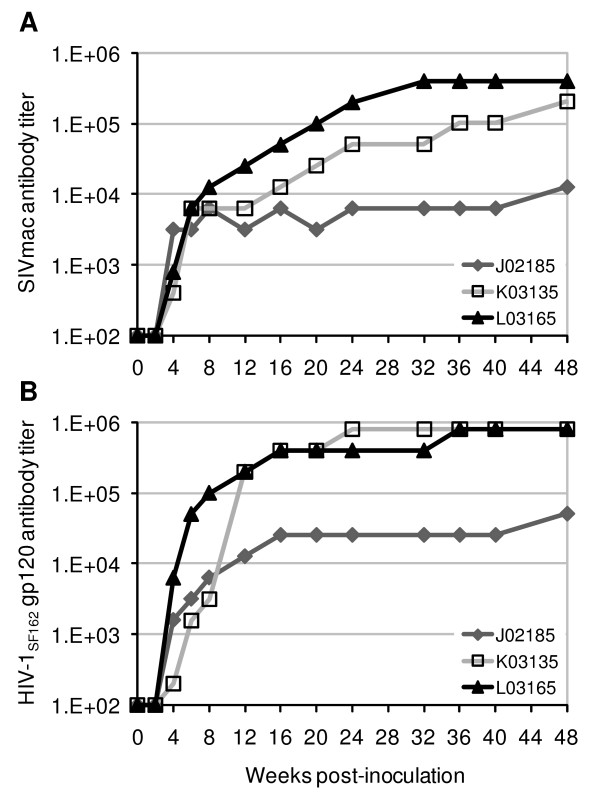
**Virus-specific antibody responses in pig-tailed macaques infected with SHIV-1157ipd3N4**. Macaque sera were collected at the indicated times following SHIV-C inoculation and analyzed by ELISA for antibody responses directed against SIVmac whole virus (A) or HIV-1_SF162 _gp120 (B). Endpoint titers were defined as the reciprocal of the highest dilution that gave an optical absorbance value at least threefold higher than the average values obtained with SIV-negative macaque sera.

All three animals that were followed beyond the acute phase of SHIV-1157ipd3N4 infection were monitored for their antigen-specific antibody responses. As shown in Fig. 6, SIV-specific (Fig. 6A) and HIV-1 gp120-specific antibodies (Fig. 6B) were detected as early as 4 wk p.i. The antibody titers continued to increase for the following 6–8 months and persisted throughout the study period of a year, including animal K03135 that showed significant peripheral blood CD4^+ ^T-cell depletion after wk 24 p.i. (Fig. 4A), as well as animal J02185 that controlled virus replication after the acute phase and showed the lowest antibody response (Fig. 1A).

Neutralizing antibody (NtAb) activity was determined by the pseudotyped virus assay in TZM-bl cells [72]. Cross-clade NtAb responses were detected as early as 24 weeks p.i. (data not shown) against both subtype A and B primary isolates (Table 1), consistent with a previous report of rhesus monkeys infected with SHIV-1157ip, the parental virus of SHIV-1157ipd3N4 [73]. Similar to antibody responses measured by ELISA, animals with high and persistent viral loads (K03135 and L03165) showed greater levels of NtAb activities, consistent with the role of antigen load driving antibody responses. It should be noted however that the neutralizing activities observed were effective only against primary viruses that were relatively easy to neutralize (Q461d1, SF162, and SS1196.1). No activity was detected against 89.6 and two viruses represented in the standard subtype B primary isolate panel (QH0692.42 and SC422661.8) [74]. No neutralization was observed against the homologous virus (data not shown) or a heterologous subtype C virus 1084i [75] (Table 1). This was in contrast to the high-titered neutralizing activity against both homologous and heterologous R5 SHIV-C isolates in rhesus macaques infected with the parental virus SHIV-1157ip [73]. Thus, the NtAb profile in SHIV-1157ipd3N4-infected pig-tailed macaques does not conform to the paradigm that homologous NtAbs arise before cross-clade NtAbs. In fact, cross-subtype NtAbs at titers similar to that in pooled plasma from subtype C-infected humans were observed in K03165 and L03165 relatively early after infection (wk 24, data not shown; and Table 1). The basis for this difference in NtAb responses is not known; but, it is likely to be due to the accessibility of conserved NtAb targets in certain isolates, regardless of their subtype classifications [76].

**Table 1:** Serum neutralizing antibody response in pig-tailed macaques 36 weeks post-inoculation with SHIV-1157ipd3N4.

**Sample**	**Q461d1 (A)^*a*^**	**SF162 (B)**	**SS1196.1 (B)**	**89.6 (B)**	**QH0692.42(B)**	**SC422661.8 (B)**	**1084i (C)**
**J02185**	<30^*b*^	67	<30	<30	<30	<30	<30

**K03135**	2,338	3,369	309	<30	<30	<30	<30

**L03165**	1,009	2,430	221	<30	<30	<30	<30

**Clade C Human**	3,060	2,795	791	743	371	NT^*c*^	812

^*a *^Subtype of the indicator viruses.

^*b *^Neutralization titers are reported as IC_50_, or the highest serum dilution at which infectivity of the indicator viruses, as measured by relative luciferase units in the TZM-bl cell assay, was reduced by 50%.

^*c *^Not tested.

## Conclusion

Our prospective analysis of SHIV-1157idp3N4-infected animals with emphasis on early acute infection demonstrates that R5 SHIV-C-induced pathogenesis in pig-tailed macaques parallel findings in CCR5-tropic SIV/SHIV rhesus models. Findings reported here support the value of pig-tailed macaques as a relevant animal model for the study of lentiviral pathogenesis and preclinical AIDS vaccines.

## Methods

### Animals

Four juvenile pig-tailed macaques (*M. nemestrina*), all negative for simian type D retrovirus by serology and polymerase chain reaction (PCR), were used in this study. Animals were inoculated intrarectally, and tissue samples were collected at specific timepoints pre- and post-inoculation for prospective monitoring of viral loads, T-cell subsets, blood chemistry, and antibody responses. General health of the animals, including body weight and temperature, was monitored by routine examinations. All animals were cared for in accordance with established National Institutes of Health guidelines, and the experimental procedures were performed with the approval of the Institutional Animal Care and Use Committee at the University of Washington.

### Virus stock

The derivation of SHIV-1157ipd3N4 was described by Song et al. [58]. A rhesus PBMC-grown stock was used for this study. All animals received 1 ml of undiluted SHIV-1157ipd3N4 by an atraumatic intrarectal inoculation. The virus stock had a p27 concentration of 95 ng/ml and an in vitro infectivity as determined by 50% tissue culture infectious doses (TCID_50_) of 10^6 ^per ml as titrated in TZM-bl cells (R. Song, personal communication).

### Cell line

TZM-bl cells (also called JC53-bl, clone 13) were contributed by John Kappes and Xiaoyun Wu and obtained from the National Institutes of Health (NIH) AIDS Reference Reagent Repository Program (catalog no. 8129) [77]. Cells were cultured in Dulbecco modified Eagle medium supplemented with 10% fetal bovine serum (FBS) and 2 mM glutamine.

### Tissue collection and isolation of lymphocytes

Peripheral blood was drawn by venipuncture into EDTA or SST tubes for extraction of plasma and PBMC, or serum, respectively. BAL samples were obtained by laryngoscope-aided introduction of a 5 mm video bronchoscope through the mouth and into the trachea. Lavage with 3–5 separate 10-ml aliquots of sterile saline was performed by injecting the saline into a terminal bronchiole, after which the saline infusate was aspirated with continuous flow vacuum at approximately 90–100 mm Hg of negative pressure. For endoscope-guided pinch biopsies obtained from the duodenum or colon, an 8.9 mm diameter video gastroscope (Karl Storz Veterinary Endoscopy, Goleta, CA) was inserted as far as the distal duodenum/proximal jejunum (90–130 cm from the mouth), or into the descending/transverse colon (50–70 cm from the anus), respectively. A maximum of 23 pinch biopsies (about 1 mm^3^) was collected via the gastroscope with sterile 2.0 mm biopsy forceps.

EDTA-treated blood was subjected to Lymphoprep (Ficoll) density gradient centrifugation for isolation of viable PBMC, or stained by a whole blood lysis technique as described below. BAL samples were centrifuged to pellet cells, but enrichment of lymphocytes was not performed. Biopsies were pooled from the duodenum or colon, treated with 5 mM EDTA and 60 U/ml collagenase, and isolated cells were enriched for lymphocytes by Percoll density gradient centrifugation, as previously described [40,42]. Viability of intestinal lymphocytes averaged 88 ± 4%, as determined by trypan blue exclusion.

### Plasma and cell-associated viral loads

Viral load was assayed as previously described [72,78]. Briefly, viral RNA load in EDTA-anticoagulated, cell-free plasma was determined by real-time RT-PCR after reverse transcriptase reaction. Proviral cDNA load in total mononuclear cells from peripheral blood or duodenal biopsies was determined by real-time PCR analysis.

### Lymphocyte immunophenotyping

Cells were stained for four-color flow cytometric analysis, using antibodies directly conjugated to either fluorescein isothiocyanate (FITC), phycoerythrin (PE), peridinin chlorophyll protein (PerCP), or allophycocyanin (APC). Briefly, for whole blood staining, 1 ml of whole blood was treated with 14 ml of ammonium chloride lysis solution for 7 min, centrifuged (700 × *g*, 5 min), and the resultant cell pellet was resuspended in 1 ml of staining medium (RPMI supplemented with 1% FBS and 0.02% NaN_3_) [72]. Fifty-μl aliquots of the cell suspension were triple-stained with CD3-FITC (SP34-2), CD4-PerCP-Cy5.5 (L200), and CD8-APC (SK1), and double-stained with CD2-FITC (S5.2) and CD20-PerCP-Cy5.5 (L27). Cells were incubated in the dark for 20 to 30 min, washed, and resuspended in 1% paraformaldehyde. For mucosal and peripheral blood lymphocytes, cells were stained by incubating 1–5 × 10^5 ^cells with combinations of monoclonal antibodies at 4°C for 30 min, washed, and then fixed in 2% paraformaldehyde. Monoclonal antibodies used were: CD3-FITC (SP34), CD3-PerCP (SP34-2), CD4-APC (L200), CD8-PerCP (SK1), CD8-APC (SK1), CCR5-PE (3A9), CD28-PE (CD28.2), and CD95-FITC (DX2). All antibodies were purchased from Becton Dickinson Biosciences (San Jose, CA). Controls included appropriate unstained, fluorescence-minus-one (FMO)-stained, and single-color-stained, samples, for compensation and gating. Data were acquired on a FACSCalibur flow cytometer (Becton Dickinson Immunocytometry Systems), where 10,000–20,000 gated lymphocyte events were collected, and analyzed with FlowJo software (Version 7.1.3, Tree Star, Ashland, OR). The absolute numbers of CD3^+^CD4^+ ^lymphocytes were determined using flow cytometry analysis software according to guidelines from the Centers for Disease Control and Prevention for T-cell determinations in HIV-infected individuals [72].

### Enzyme-linked immunosorbent assay (ELISA)

The titers of whole virus SIVmac- and HIV-1_SF162 _gp120-specific antibodies were measured by ELISA as previously described [79]. Endpoint titers were determined as the reciprocal of the highest serum dilution that resulted in an optical density reading greater than the average values obtained with negative macaque sera plus three standard deviations.

### Neutralization assay

The neutralization activity of sera from SHIV-1157ipd3N4-inoculated pig-tailed macaques was measured in a pseudotyped virus assay as described [72]. Indicator viruses with envelope derived from the following HIV-1 were used: subtype A isolate Q461d1 [80]; subtype B isolates SF162, SS1196.1, 89.6, QH0692.42, and SC422661.8 [72,74]; and subtype C isolate 1084i [75]. All assay stocks were titrated in TZM-bl cells as described previously [72,74]. Indicator virus containing 150 TCID_50 _was incubated with serial dilutions of serum samples (starting at 1:30) in triplicate in a total volume of 60 μl for 1.5 hr at 37°C in 96-well U-bottom tissue culture plates (Corning). TZM-bl cells plated 24 hr previously (3,000 cells in 100 μl of growth medium) were treated with 2 μg/ml polybrene for 30 min at 37°C. One set of control wells received cells plus virus (virus control), and another set received cells only (background control). After a 72-hr incubation, luciferase activities were analyzed using BrightGlo substrate solution as described by the supplier (Promega). Neutralization activity was expressed as the highest serum dilution that resulted in 50% reduction of relative luciferase units (RLU). Values obtained with pre-inoculation sera were subtracted from those obtained with post-inoculation sera for each animal.

## Competing interests

The authors declare that they have no competing interests.

## Authors' contributions

OH, KL, RMR, RS, and SLH designed the study. RMR and RS provided the SHIV-1157ipd3N4 virus stock. DA and SLH established funding for the study. KL coordinated and performed the primate studies. OH performed sample processing and immunophenotyping experiments. PP coordinated the viral load and ELISA experiments. YL performed the neutralization experiments. OH, PP, YL, and SLH analyzed the data, and OH and SLH wrote the manuscript. All authors read and approved the manuscript.
